# Characteristics of flight delays during solar flares

**DOI:** 10.1038/s41598-023-33306-9

**Published:** 2023-04-13

**Authors:** X. H. Xu, Y. Wang, F. S. Wei, X. S. Feng, M. H. Bo, H. W. Tang, D. S. Wang, L. Bian, B. Y. Wang, W. Y. Zhang, Y. S. Huang, Z. Li, J. P. Guo, P. B. Zuo, C. W. Jiang, X. J. Xu, Z. L. Zhou, P. Zou

**Affiliations:** 1 Shenzhen Key Laboratory of Numerical Prediction for Space Storm, Institute of Space Science and Applied Technology, Harbin Institute of Technology, Shenzhen, China; 2 Key Laboratory of Solar Activity and Space Weather, National Space Science Center, Chinese Academy of Sciences, Beijing, China; 3 Travelsky Mobile Technology Limited, Beijing, China; 4 Nanjing University of Information Science and Technology, Nanjing, China; 5 Institute for Frontiers in Astronomy and Astrophysics, Beijing Normal University, Beijing, China; 6 State Key Laboratory of Lunar and Planetary Sciences, Macau University of Science and Technology, Macao, China; 7 Planetary and Space Physics Group, Department of Astronomy, Beijing Normal University, Beijing, China

**Keywords:** Solar physics, Space physics

## Abstract

Solar flares are one of the severest solar activities that have important effects on near-Earth space. Previous studies have shown that flight arrival delays increase as a result of solar flares, but the intrinsic mechanism behind this relationship is still unknown. In this study, we conducted a comprehensive analysis of flight departure delays during 57 solar X-ray events by using a huge amount of flight data (~ 5 × 10^6^ records) gathered over a 5-year period. It is found that the average flight departure delay time during solar X-ray events increased by 20.68% (7.67 min) compared to quiet periods. Our analysis also revealed apparent time and latitude dependencies, with flight delays being more serious on the dayside than on the nightside and longer (shorter) delays tending to occur in lower (higher) latitude airports during solar X-ray events. Furthermore, our results suggest that the intensity of solar flares (soft X-ray flux) and the Solar Zenith Angle directly modulate flight departure delay time and delay rate. These results indicate that communication interferences caused by solar flares directly affect flight departure delays. This work expands our conventional understanding of the impacts of solar flares on human society and provides new insights for preventing or coping with flight delays.

## Introduction

Solar flares are among the severest solar phenomena, characterized by the sudden and intense release of electromagnetic energy from the sun^1^. They have significant impacts on the near-Earth space environment and are therefore an important subject of space weather research^2,3^. It has been widely recognized that solar flares can adversely affect various systems, including spacecraft operations, high-frequency (HF) radio communications^4^, GPS/GNSS navigation^5^, and air traffic control facilities^6^. With the development of modern science and technology, the impacts of solar flares on human society have been found to be more and more obvious. A solar flare is a sudden and intense release of energy (about 10^28^–10^32^ ergs)^7–9^ from the sun, which typically occurs within a matter of minutes to a few hours and can emit various forms of electromagnetic radiation, most notably X-rays and extreme ultraviolet^10^. These radiations can induce a rapid increase in ionization within the Earth's lower ionosphere, specifically the D region^11,12^, resulting in a series of ionospheric disturbances that can cause fading or even disruption of HF radio signals, interfering with communication and navigation systems. However aircraft communication must be available throughout the entire flight route to ensure safety^13^. Therefore, the impact of solar flares on commercial flights cannot be overlooked.

There have been sporadic reports of solar flares affecting flight operations. For example, in November 2015, air traffic control radar stations in Sweden experienced disruptions caused by solar flare-associated radio radiations, resulting in delays in Swedish airspace that lasted for nearly an hour^6,14^. Similarly, in September 2017, a series of fairly strong solar flares caused periodic impacts on HF radio users globally. The French Civil Aviation authorities reported that an aircraft equipped with non-Controller Pilot Data Link Communications equipment lost HF radio contact for approximately 90 minutes^15^. Additionally, some researchers found that the number of aviation catastrophes increased on the third day following an X-class solar flare^16^.

The impacts of solar flares on the aviation industry have been researched by various international organizations and scholars^17,18^. However, their focus has primarily been on safety concerns, with little attention paid to flight delays, which are a matter of significant interest to many passengers. Previous research has clearly demonstrated that flight delays can lead to significant economic losses and generate a lot of issues^19,20^. Therefore, there is a crucial need to investigate the relationship between solar flares and flight delays. Our previous research has suggested that flare-induced magnetospheric-ionospheric disturbances are strongly linked to flight delays^21^. Nevertheless, what are the distinctive characteristics of flight delays during solar flares? And what is the underlying mechanism that connects flight delays with solar flares? In this research, based on the data of solar flares from GOES satellite and huge amounts (~ 5 × 10^6^) of flight records, the time and latitude dependency of flight delays during solar flares are revealed and the relevant potential explanations are discussed. It should be noted that our investigation on solar flares only takes into account the electromagnetic radiation emitted from the sun, but does not consider the high-energy particles that often accompany them.

## Data and methods

**Figure 1:**
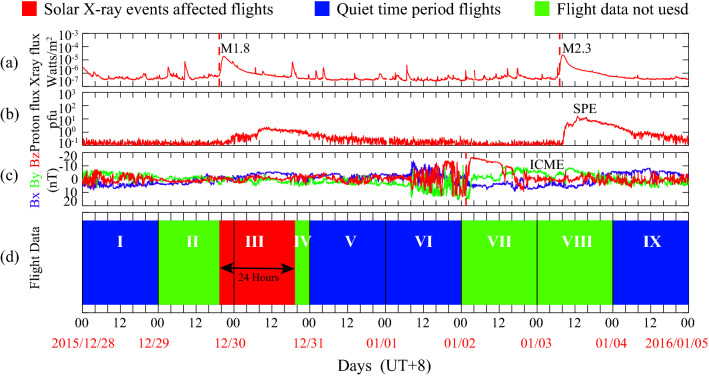
The definition of solar X-ray events affected flights (red) and quiet time periods flights (blue). (**a**) Solar soft X-ray flux from the GOES satellite. (**b**) Solar magnetic component flux from the ACE satellite. (**c**) Proton flux from the GOES satellite. (**d**) Flight data based on scheduled departure time.

Solar X-ray events are defined by soft X-ray (1–8 Å) fluxes from the GOES satellites, and they can be obtained from the National Oceanic and Atmospheric Administration (NOAA) Space Weather Prediction Center (SWPC). The details for determining the begin, maximum, and end times of an X-ray event can be found on the NOAA website^22^or in the relevant literature^23^. The list of solar X-ray events used in this paper is obtained from NOAA^24^, while unlisted events are identified by using the SWPC algorithm based on soft X-ray data obtained from the GOES satellites. According to the NOAA Space Weather Scales, solar X-ray events below class M typically do not significantly affect communications and navigation. Therefore, only M-class and X-class solar X-ray events are selected for this analysis. In this work, only independent solar X-ray events, such as the M1.8 class solar X-ray event shown in Fig. 1a, are chosen as research samples, while solar X-ray events that partially overlapped with other space weather events (e.g., coronal mass ejections or solar proton events), such as the M2.3 class solar X-ray event shown in Fig. 1a, are excluded. The begin and end times of a coronal mass ejection can be obtained from the website^25^, and the begin and maximum times of a solar proton event can also be obtained from database^26^. The end time of a solar proton event is defined as the time at which the proton flux of the GOES satellite drops below 10 pfu.

The individual flight data for this study were obtained from Travelsky Mobile Technology Limited, an affiliated company of the Civil Aviation Administration of China. The dataset contains information on operational dates, flight numbers, departure and arrival airports, as well as scheduled and actual departure and arrival times for all commercial flights between January 1st, 2015 and December 30th, 2019. The airports included in the dataset are Shenzhen Baoan International Airport (IATA: SZX) (22.64°N, 113.82°E), Guangzhou Baiyun International Airport (IATA: CAN) (23.38°N, 113.30°E), Shanghai Pudong International Airport (IATA: PVG) (31.15°N, 121.80°E), Shanghai Hongqiao International Airport (IATA: SHA) (31.19°N, 121.33°E) and Beijing Capital International Airport (IATA: PEK) (40.07°N, 116.58°E). These airports are ideal samples for analyses since they are the five largest hub airports in China, which could statistically guarantee the data homogeneity.

The present study focuses on the departure delay as the research object, which is defined as the time difference between the actual departure time and the scheduled departure time of a flight. It has been previously demonstrated that there exists a linear correlation between the arrival delay and departure delay^27,28^. In addition, our previous study^21^ has examined the effect of space weather on arrival delays, which can be contrasted with the present work. Thus, the focus of the current analysis is on the departure delay rather than the arrival delay.

It is important to note that flight delays exhibit intrinsic characteristics, including being influenced by various contingencies and having inherent periods. Therefore, to accurately determine the impact of solar flares on flight delays, these factors need to be carefully considered. Flight delays are highly complex, nonlinear, and interrelated, so the research samples should be large enough to smear out those various contingencies as much as possible. To eliminate the influence of accidents, a comparative study similar to Mitsokapas et al*.*^29^ was adopted, but with a larger research sample size (~ 5 × 10^6^ records over 5 years). It is worth noting that the selected solar X-ray events were randomly distributed across weekdays and months, thereby minimizing the influence of weekday and season effects on the results^21^. Nevertheless, the fact that flight delays exhibit a 24-h intrinsic period is one of the most important factors considered in this study^21^. The “24-h intrinsic period” is that flight delays vary significantly at different moments of the day^30–32^. To eliminate such influence, flights departure within the 24-h-period after the onset of the solar X-ray events are defined as the solar X-ray events affected flights, as marked in red (III) and shown in Fig. 1d. Correspondingly, we excluded the nearby flight data (II and IV) since they did not span an entire day (from 00:00 LT to 24:00 LT), as illustrated in green in Fig. 1d. In addition, we excluded scheduled departure flights during coronal mass ejections or solar proton events (VII and VIII), as indicated in green in Fig. 1d. Moreover, if multiple solar X-ray events occur within 24 h, we only take into account the flight data for the first solar X-ray event within the selected range. Finally, scheduled departure flights that were not disrupted by solar X-ray events, coronal mass ejections, or solar proton events are retained as flight data for quiet periods (I, V, VI and IX), as highlighted in blue in Fig. 1d.

For the analysis in this study, a total of 52 solar X-ray events and 5,295,347 individual flight records (1,751,250 records are filtered out) were selected from January 1st, 2015 to December 30th, 2019. It should be noted that all the cancelled flights are removed from our dataset for the entire study, since the percentage of such flights (86,997 records) is too small to impact the final results.

## Results

Solar flares can have varied effects on Earth, depending on several factors such as the local time (geographical longitude), geographical latitude, flare intensity, and Solar Zenith Angle (SZA)^33–35^. If the increased flight delay time during solar X-ray events is due to solar flares rather than statistical coincidences, the delay time should reveal some distinct features related to solar flares. Identifying such features would facilitate a better understanding of the causal relationship between solar flares and flight delays.

**Figure 2:**
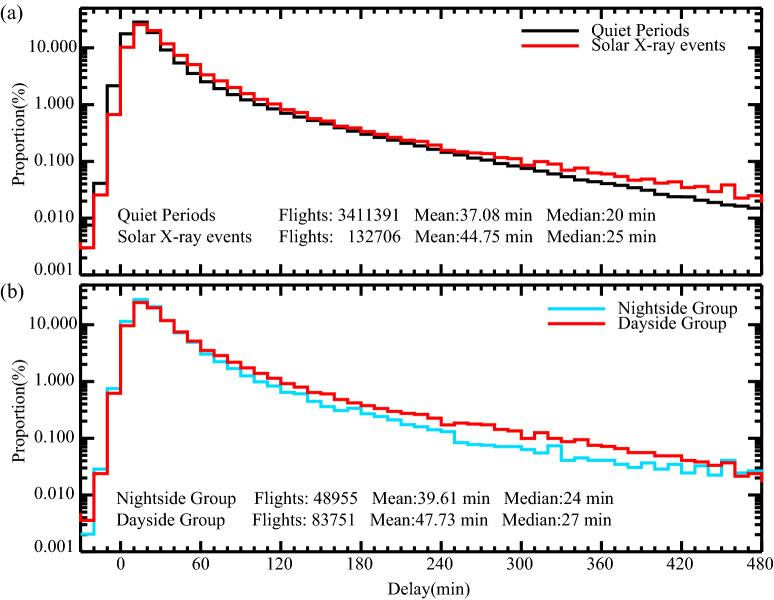
(**a**) The PDF of flight departure delay time during solar X-ray events (red) and quiet periods (black) averaged over five airports. (**b**) The PDF of flight departure delay time in the “Dayside Group” (red) and “Nightside Group” (blue) averaged over five airports.

To comprehensively assess the impacts of solar X-ray events on flight departure delays, it is essential to compute the empirical probability density function (PDF) of flight departure delays based on all individual flight data in all airports, as shown in Fig. 2a. Our analysis reveals that flight delays statistics during solar X-ray events are shifted to the right compared to quiet periods, indicating an overall larger delay time. The average delay time during solar X-ray events is 44.75 min (median: 25 min), which is 7.67 min (median: 5 min) longer than during quiet periods. These findings demonstrate that flight departure delays will increase during solar X-ray events, consistent with our previous study on arrival delays^21^.

The longitudinal effect (local time) is a key factor that affects the Earth's response to solar flares^33^. Theoretically, the impact of solar flares on the Earth is mainly concentrated in the subsolar point and varies smoothly with the cosine of SZA^36,37^. It can be inferred that during solar flares, HF radio absorption will be more prominent in the dayside ionosphere compared to the nightside ionosphere, where little absorption is expected. Consequently, solar flares can severely disrupt communications and navigation on the dayside, while having little effect on the nightside. However, in this study, the longitudinal variations among the airports are minor, which makes it difficult to directly investigate the longitudinal effect of the flare on flight delays. To reveal the longitudinal effect, all the solar X-ray events affected flights were further categorized into two groups based on the onset of the solar X-ray events: the "Dayside Group" included events that began between 06:00 (LT) and 18:00 (LT), and the remaining events comprised the "Nightside Group". It is worth noting that, no matter when solar X-ray events occur, each group covers all flight data for a full 24-h-period, and it was crucial in removing the hourly delay difference introduced by the “24-h intrinsic period”^30–32^.

Figure 2b illustrates the PDF of flight departure delays during 33 solar X-ray events in the "Dayside Group" and 19 solar X-ray events in the "Nightside Group". Two obvious characteristics can be observed in the figure. Firstly, the average flight delay time during solar X-ray events, regardless in nightside group or dayside group, is larger than that during quiet periods. As solar X-ray events can last for several hours and aviation activities require time to respond, such circumstances may partially account for the increased delay time for the nightside group. Secondly, the delay statistics for the dayside group exhibit a rightward shift compared to those for the nightside group, indicating larger overall delays. These results confirm our expectation that flight delays in the dayside group were more severe than those in the nightside group. Therefore, the above results also suggest that the solar X-ray events affected flight delays reveal obvious time dependency.

Finally, it is noteworthy that latitude is also an important factor that controls the effects of solar flares on Earth^34,36^. As the investigated airports are located in similar longitudes but varying in latitudes, they provide an excellent opportunity to examine the latitude effect. Based on their geographical coordinates, the five airports were classified into three categories: the higher latitude airport, PEK; the middle latitude airport, SHA and PVG; and the lower latitude airport, CAN and SZX. It has been known that solar flares tend to have a more pronounced impact on regions closer to the equator, in contrast to higher latitudes, as revealed by previous research^38,39^. The adverse effects of solar flares on various communication and navigation systems are generally more severe in lower latitude areas. This feature is expected to result in comparatively longer flight delays in lower latitude airport and shorter delays in higher latitude airport.

**Figure 3:**
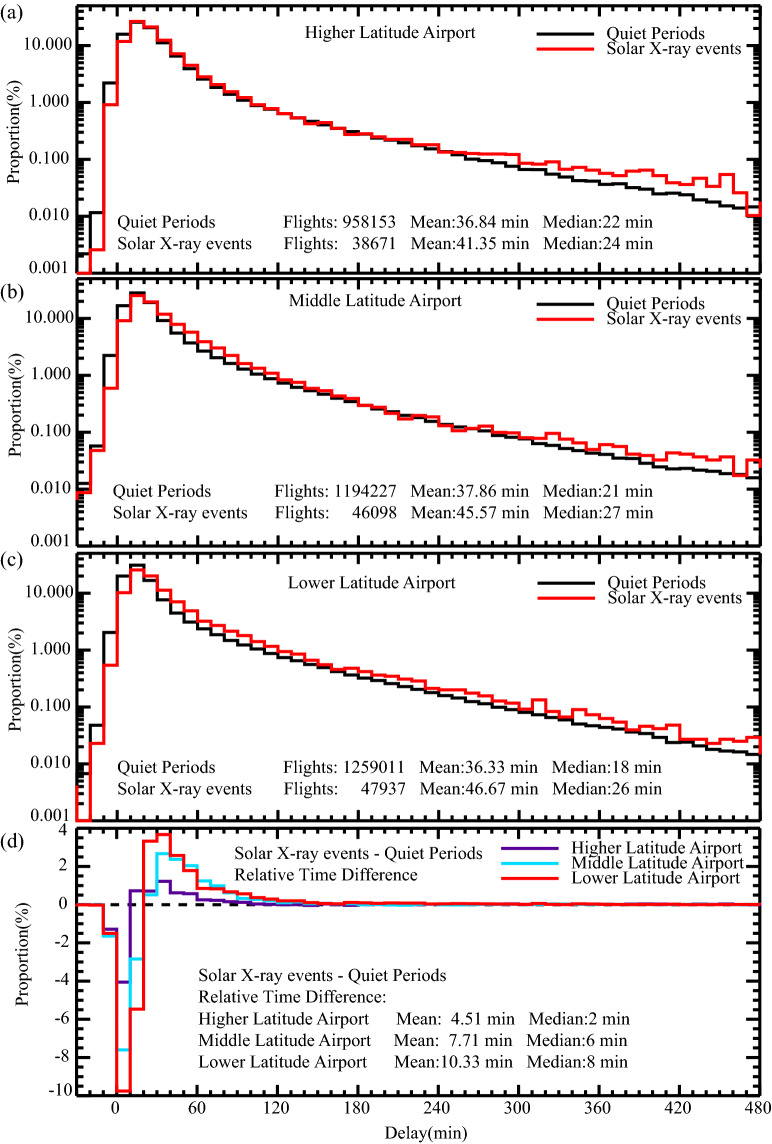
The PDF of flight departure delay time during solar X-ray events (red) and quiet periods (black) at higher (**a**), middle (**b**), and lower (**c**) latitude airport respectively. (**d**) Distributions of the relative difference of delay time between those during solar X-ray events and quiet periods (higher latitude airport: purple, middle latitude airport: blue, lower latitude airport: red).

The departure delay PDF for three types of airports during both quiet periods and solar X-ray events are illustrated in Fig. 3a, b, and c. It is evident that the flight average delay time during solar X-ray events decreases from lower to higher latitude airports with values of 46.67, 45.57, and 41.35 min (with median values of 26, 27, 24 min), respectively. Meanwhile, the average delays during quiet periods remain nearly unchanged at ~ 37 min. The latitude effect of flight delays is clearly revealed, as the lower latitude airports consistently exhibit higher delays. The relative difference in delay time between the solar X-ray events and quiet periods, as presented in Fig. 3d, can be interpreted as the net increase in delay time caused by solar X-ray events. Notably, the net increase in average delay time also shows a smooth change with latitude (10.33 min near ~ 23°N, 7.71 min near ~ 31°N and 4.51 min near ~ 40°N), and the change rate is approximately 0.35 min/degree. These findings provide compelling evidence that the impact of solar X-ray events on flight delays varies significantly by latitude, which is also consistent with expectations.

## Discussions and conclusions

It is crucial to eliminate various interference factors to obtain statistically valid results on the effect of solar flares on flight delays. Although we have removed the influence of additional major space weather events, such as coronal mass ejections and solar proton events, there may still be some measurable solar effects that could contaminate the "quiet periods" in our analyses. However, these "contaminations" can only underestimate the impact of solar flares on flight delays^21^, which does not change the overall conclusions. Besides, to ensure that there are indeed differences in flight delays, we conducted the two-sample Kolmogorov–Smirnov test on the "quiet periods" and various "solar X-ray event periods" shown in Figs. 2 and 3, respectively. All the calculated critical values indicate that the distributions of the referred flight delays during "quiet periods" and various "solar X-ray event periods" are indeed different, with a significance level of 0.05.

Our previous study has indicated that solar flares can have an impact on flight arrival delays, which are highly correlated with magnetospheric-ionospheric disturbances^21^. Our findings suggest that departure delays associated with solar X-ray events are also similar to arrival delays. Furthermore, the observed time and latitude dependency of departure delay time strongly implies that there is a causal relationship between solar X-ray events and flight delays, rather than just coincidental effects. To investigate the underlying mechanism that links flight delays and solar flares, we will focus on the intensity of the solar flares and the SZA.

The intensity of solar flares, represented by the soft X-ray flux, is a critical factor in determining their effects on Earth. It is known that the flare associated effects on Earth would be more significant if the flux intensity is more intense. Moreover, the effects of solar flares on Earth are not solely dependent on the X-ray flux but also on the SZA. Many researchers have considered the influence of SZA when investigating the impacts of solar flares on human activities^34,40^. The DRAP model^36^ is often used to estimate flare-associated HF radio absorption, and the Highest Affected Frequency (HAF) is introduced to determine the highest frequency affected by the absorption of 1 dB due to solar X-ray flux^41^.

$$HAF = \left[ {10\log \left( {flux} \right) + 65} \right](\cos \chi )^{0.75}$$

where the *flux* represents the soft X-ray flux and χ is the SZA. The HAF is an empirical formula that can be used to simply evaluate the interferences of HF communication caused by solar flares. Obviously, the higher X-ray flux and the smaller SZA will lead to more significant consequences.

**Figure 4:**
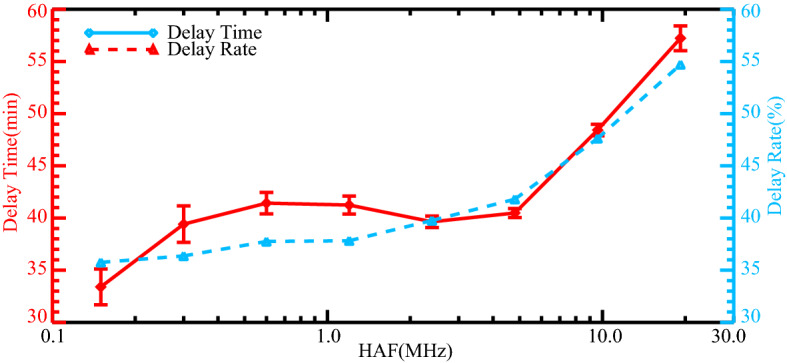
The flight departure delay time (red) and delay rate (blue) as a function of HAF during solar X-ray events averaged over five airports. The vertical error bars represent the standard error.

Figure 4 shows the flight departure delay time and delay rate as a function of HAF during solar X-ray events, averaged over five airports. It is observed that the delay time and delay rate exhibit a rough increase as the HAF value increases. When the HAF is relatively small, gentle increases or fluctuations in flight delays are found. However, the most notable finding is that the growth rate of both the delay time and delay rate are increased dramatically when the HAF exceeds 5 MHz. This result further verifies the effects of solar X-ray events on flight delays and suggests that solar X-ray events associated interferences in HF communication should be the most probable reason accounting for the increased flight delays. In summary, this study examines the unique characteristics of flight delays during solar X-ray events and aims to establish the underlying relationships between them. The results reveal that the average departure delay time for flights in all airports during solar X-ray events is 7.67 min (20.68%) longer compared to those during quiet periods. The study also identified a time dependency, with longer (shorter) flight delays occurring at the dayside (nightside) during solar X-ray events. Furthermore, our analysis identifies a latitude dependency, whereby lower (higher) latitude airports experience longer (shorter) delays during solar X-ray events. We also investigate the factors that contribute to flight delays during solar X-ray events by examining the relationship between the HAF and flight delays. It is indicated that both the soft X-ray flux and the SZA play important roles in flight delays. These findings demonstrate that communication interferences caused by solar X-ray events directly affect flight departure delays. This work extends our conventional understanding of solar flares, and it could also provide us with brand new views to help improve the flight delay predications.

## Data Availability

The intact flight data that used in this study are available from Travelsky Mobile Technology Limited but restrictions apply to the availability of these data, which were used under license for the current study, and so are not publicly available. The data are however available from the authors upon reasonable request and with permission of Civil Aviation Administration of China.
